# Dietary Plant Sterols Supplementation Increases *In Vivo* Nitrite and Nitrate Production in Healthy Adults: A Randomized, Controlled Study

**DOI:** 10.1111/1750-3841.13752

**Published:** 2017-07-14

**Authors:** Xing Lin Ho, Wai Mun Loke

**Affiliations:** ^1^ Centre for Functional Foods & Human Nutrition, School of Chemical & Life Sciences Nanyang Polytechnic 180 Ang Mo Kio Ave 8 Singapore 569830 Singapore; ^2^ Food Science & Nutrition Group, School of Chemical & Life Sciences Nanyang Polytechnic 180 Ang Mo Kio Ave 8 Singapore 569830 Singapore

**Keywords:** L‐arginine, nitrate, nitric oxide, nitrite, plant sterols

## Abstract

A randomized, double‐blinded, placebo‐controlled and crossover study was conducted to simultaneously measure the effects, 3 h after consumption and after 4‐wk daily exposure to plant sterols‐enriched food product, on *in vivo* nitrite and nitrate production in healthy adults. Eighteen healthy participants (67% female, 35.3 [mean] ± 9.5 [SD] years, mean body mass index 22.8 kg/m^2^) received 2 soy milk (20 g) treatments daily: placebo and one containing 2.0 g free plant sterols equivalent of their palmityl esters (β‐sitosterol, 55%; campesterol, 29%; and stigmasterol, 23%). Nitrite and nitrate concentrations were measured in the blood plasma and urine, using stable isotope‐labeled gas chromatography‐mass spectrometry. L‐arginine and asymmetric dimethylarginine concentrations in blood serum were measured using commercially available enzyme immunoassays. Nitrite and nitrate concentrations in blood plasma (nitrite 5.83 ± 0.50 vs. 4.52 ± 0.27; nitrate 15.78 ± 0.96 vs. 13.43 ± 0.81 μmol/L) and urine (nitrite 1.12 ± 0.22 vs. 0.92 ± 0.36, nitrate 12.23 ± 1.15 vs. 9.71 ± 2.04 μmol/L) were significantly elevated after 4‐wk plant sterols supplementation Placebo and 3‐h treatments did not affect the blood plasma and urinary concentrations of nitrite and nitrate. Circulating levels of L‐arginine and asymmetric dimethylarginine were unchanged in the placebo and treatment arms. Total plant sterols, β‐Sitosterol, campesterol, and stigmasterol concentrations were significantly elevated after 4‐wk treatments compared to the placebo and 3‐h treatments. Blood plasma nitrite and nitrate concentrations correlated significantly with the plasma total and specific plant sterol concentrations. Our results suggest that dietary plant sterols, in the combination used, can upregulate nitrite, and nitrate production *in vivo*.

## Practical Application

Plant sterols supplementation in the presence of a food matrix may upregulate the vascular nitric oxide expression, and contribute to vascular health. Specific plant sterols, such as β‐sitosterol, campesterol, and stigmasterol exerted differential effects on the vascular nitric oxide production. The incorporation or enrichment of plant sterols mixture or specific plant sterols, like campesterol, into suitable food products, may enhance their vascular health benefits.

## Introduction

Inflammation, oxidative stress, alterations in hemodynamic forces, and other injurious stimuli may cause vascular endothelial cells to exhibit proatherogenic alterations (Verma and Anderson 2002). Endothelial function has been proposed to serve as an excellent “barometer” of underlying vascular health (Vita and Keaney 2002), and modulation of endothelial function may serve as a potential cardiovascular therapeutic target. It is agreed that endothelial change can be documented by measuring plasma levels of endothelial cell‐generated vaso‐reactive molecules, like nitric oxide (NO). The vascular endothelium maintains vascular homeostasis and regulates vascular tone by controlling the production of the vasodilating NO (Rassaf and others 2002). The endothelial nitric oxide synthase (eNOS) catalyzes the formation of NO from L*‐*arginine in the presence of NADPH and oxygen (Rassaf and others 2002). The NO production *in vivo* has been shown to be increased after L*‐*arginine oral supplementation (Clarkson and others 1996; Thorne and others 1998). The changes in the eNOS competitive inhibitor, asymmetric dimethyl arginine (ADMA), levels would also affect the *in vivo* NO production (Nagase and others 1997; Maxwell and Cooke 1998). Short‐lived NO is oxidized to nitrite and nitrate in the vaculature (Gladwin and others 2006). The circulating nitrite and nitrate serve as a reservoir for NO *in vivo* as these molecules can be reduced back to NO in the vasculature if so required to maintain vascular tone (Gladwin and others 2006). Measuring the circulating nitrite and nitrate concentrations may be useful to indicate eNOS activity (Kleinbongard and others 2003).

A diet rich in fruits and vegetables is associated with a lower risk of cardiovascular disease, but the mechanisms behind this protection are not completely understood (Hu and Willett 2002). These beneficial effects may be related to the presence of specific phytochemicals in the fruits and vegetables, such as plant sterols (PS). The circulating concentrations of PS are dependent on the diet and absorption efficiency, as these molecules are not endogenously synthesized by humans (Ling and Jones 1995). The intake of PS from diet ranges from 150 to 400 mg/d, with 65% as β‐sitosterol, 30% as campesterol, and 5% as stigmasterol (Ling and Jones 1995; Ostlund and others 2002). Regular consumption of foods containing PS has been shown to lower blood triglycerides and low‐density lipoprotein–cholesterol concentrations (Lau and others 2005; Chan and others 2007). Meta‐analyses showed reductions of blood cholesterol levels by about 9% with 2 g/d PS consumption (AbuMweis and others 2008; Demonty and others 2008). The effects of PS supplementation on endothelial function is less understood (De Jongh and others 2003; Hallikainen and others 2006; De Jong and others 2007). Even though NO bioavailability has been established to be essential to maintain vascular tone, little is known about the effects of PS on the endothelial production of NO in healthy adults.

Our study examined and compared the actions of PS, in the presence of a food matrix, on the *in vivo* NO production of healthy adults 3 h after consumption, and after 4‐wk daily exposure. We hypothesized that PS supplementation increases nitrite and nitrate production *in vivo*. A separate set of *in vitro* experiments examined the effects of specific PS, β‐sitosterol, campesterol, and stigmasterol on the nitrite and nitrate production from freshly isolated human neutrophils.

## Materials and Methods

### Chemicals and materials

Potassium hydroxide, sodium acetate, sodium hydroxide, hydrochloric acid, formic acid, glucuronidase, phorbol 12‐myristat 13‐acetate (PMA), phosphate‐buffered saline (PBS), diethylenetriamine penta‐acetic acid, fetal bovine serum (FBS), penicillin, streptomycin, bFGF, heparin, 5α‐cholestane, β‐sitosterol, stigmasterol, isooctane, [^15^N]‐sodium nitrite, [^15^N]‐sodium nitrate, 2,3,4,5,6‐pentafluorobenzylbromide (PFBBr), N,O‐bis(trimethylsilyl)trifluoroacetamide in 1% TMS (BSTFA in 1% TMS) and pyridine were purchased from Sigma–Aldrich (Mo., U.S.A.); glucose and Hank's buffered salt saline (HBSS) were purchased from Merck (VIC, Australia); acetonitrile, ethyl acetate, hexane, methanol, ethanol, acetone, and from Tedia (Ohio, U.S.A.); ficoll‐paque was from GE Healthcare (Uppsala, Sweden); and RPMI1640 were from Gibco Invitrogen (Carlsbad, Calif., U.S.A.); and dextran 500 was from Amersham Biosciences (Uppsala, Sweden); campesterol from Santa Cruz Biotechnology (Texas, U.S.A.). Soy milk powder custom‐blended with the BASF Vegapure® 67WDP [palmityl esters of *β*‐sitosterol, max 55% (w/w); campesterol, max 29% (w/w) and stigmasterol, max 23% w/w] and the corresponding placebo soy milk powder were manufactured by Medispec (M) Sdn Bhd (Malaysia). The nutritional compositions of the placebo and PS‐enriched soy milk powders were analyzed by a commercial food laboratory (TUV SUD PSB, Singapore) using accredited AOAC‐modified methods, and were found to be comparable with the exception of PS contents.

### Study design

Eighteen healthy adults of Asian ethnicities, age above 21 y, were included in this study. Exclusion criteria for recruitment include body mass index >30, alcohol consumption >20 g/d (more than 2 standard drinks per day) and medical conditions, such as diabetes mellitus, hypertension, hypercholesterolemia, and other cardiovascular‐related diseases. Pregnant women and women planning to conceive were not allowed to participate. The study participants were recruited via advertisements on the local newspaper. Clinical information [including age, gender, body mass index (BMI), medical history, weight, and height] were collected using structured questionnaires. For a 1‐wk period prior to commencement of the study, participants were told to refrain from changes from their usual dietary habits. The participants were randomized to receive the 2 treatments in different orders. The treatments corresponded to:

1. 2.0 g free PS equivalent of their palmityl esters (BASF Vegapure® 67WDP β‐sitosterol, max 55% [w/w]; campesterol, max 29% [w/w] and stigmasterol, max 23% [w/w]) in soy milk powder (20 g), dissolved in 300 mL of warm water.
2. Soy milk powder (20 g) placebo (control), dissolved in 300 mL of warm water.

On each test day, participants attended the clinic after 12 h overnight fast. A baseline set of whole blood (20 mL) and spot urine (10 mL) were collected. The assigned 1st treatment was consumed over 15 min. A 2nd set of blood and urine was taken 3 h after treatment. The 3‐h time point was chosen to examine the acute effects of the plant sterols supplementation. The same participants received on a daily basis of the first assigned treatment for a period of 28 d. At the end of the 4‐wk daily exposure period, spot whole blood, and urine were collected. The participants were subjected to 1‐wk wash‐out period before the 2nd treatment. The 1‐wk wash‐out period was sufficient for the measured parameters to return to the baseline values. Altogether, each participant reported to the clinic 4 times for the entire study. These biological samples were processed to obtain urine, blood plasma, and serum for storage at −80 °C. The participants were instructed to maintain their weight and activity level during the study, and they were counseled to refrain from changing their usual dietary habits during the study. The participants were told to return the remaining intervention material at the ends of the 2 crossover arms. The study protocol was reviewed and approved by the Institutional Review Board, Nanyang Polytechnic, Singapore, and each subject provided written informed consent prior to their study involvement.

### Circulating concentrations of nitrite, nitrate, L‐arginine, and asymmetric dimethylarginine

The vascular NO bioavailability was examined by assessing the circulating concentrations of nitrite and nitrate. The nitrite and nitrate concentrations in the blood plasma and urine were measured using stable isotope‐labeled gas chromatography ‐ mass spectrometry (GC‐MS) as previously described (Tsikas 2000). Briefly, the sample fluid was spiked with internal standards, (^15^N) sodium nitrite (6 ng), and (^15^N) sodium nitrate (40 ng). The spiked sample was derivatized with acetone and PFPBr at 50 °C for 30 min. After the removal of acetone, the remaining aqueous phase was extracted with toluene and the organic extract (0.5 L) was analyzed by using an Agilent 6890 gas chromatograph coupled to a 5973 mass spectrometer fitted with a cross‐linked methyl silicone column (25 m x 0.20 mm, 0.33 mm film thickness, HP5‐MS) by using negative‐ion chemical ionization. Samples (1.0 μL) were injected in the splitless mode, and the oven temperature was held at 70 °C for 1 min, then increased to 160 °C at a rate of 20 °C/min and finally to 280 °C at a rate of 30 °C/min. Helium (92.5 kPa and flow rate 0.7 mL/min) was used as the carrier and methane as the reagent gas for negative‐ion chemical‐ionization. Peak identification was based on retention time and mass spectra compared with ^15^N‐labeled authentic standards (sodium [^15^N] nitrite and sodium [^15^N] nitrate). Quantification was performed by using calibration curves obtained from authentic standards and labeled standards. Plasma concentrations of nitrite and nitrate were expressed unadjusted, while their urinary concentrations were adjusted for urinary creatinine levels.

The concentrations of L‐arginine and ADMA in the blood plasma were quantified using a commercial ELISA Kit (DLD Diagnostika GmbH, Hamburg, GERMANY).

### Circulating concentrations of total and specific plant sterols

The concentrations of β‐sitosterol, campesterol, and stigmasterol in blood plasma were determined using GC‐MS as previously described (Ahmida and others 2006). Briefly, internal standard (5α‐cholestane, 2 nmol) was added into blood plasma (200 μL). The mixture was hydrolyzed with ethanolic potassium hydroxide (1 mol/L, 1 mL) at 70 °C for 60 min in the dark. The lipids were extracted twice with hexane and ethanol, containing 12.5 mg/ L BHT (20:1 v/v, 1 mL). The dried lipid extract was derivatized with pyridine‐BSTFA with 1% TMCS (1:1 [v/v], 200 μL) at 70 °C for 60 min. The derivatized extract was dried under nitrogen and reconstituted in isooctane (20 μL) before injecting into the GC‐MS. The MS was operated in the electron ionization mode. The 5α‐cholestane, β‐sitosterol, campesterol, and stigmasterol were monitored at *m*/*z* 357, 486, 472, and 484, respectively. The concentrations of specific PS were calculated based on the corresponding calibration curves obtained using the respective standards.

### *In vitro* experiments

A separate set of i*n vitro* experiments was carried out to examine the specific augmentation of NO production by specific PS. The eNOS‐inducing effect of specific PS was measured by the production of nitrite and nitrate from human blood neutrophils. Briefly, human blood neutrophils were isolated from the neutrophil/erythrocyte pellet after Ficoll Paque gradient centrifugation and dextran sedimentation of red blood cells as previously described (Loke and others 2012). Cell viability was assessed using trypan blue exclusion and was typically >98%. The freshly isolated neutrophils were resuspended in HBSS at a concentration of 5 × 10^6^ cells/mL. To examine the effects of β‐sitosterol, campesterol, and stigmasterol on eNOS activity, freshly isolated human blood neutrophils (5 × 10^6^ cells/mL in HBSS, 1 mL) was incubated with either β‐sitosterol, campesterol, or stigmasterol (final concentrations, 0, 2, 5, 10, 20, and 50 μmol/L) and arginine (final concentration, 10 μmol/L) at 37 °C for 5 min prior to stimulation. The PS were added using ethanol as vehicle. The cells were stimulated with PMA (final concentration, 200 nmol/L) at 37 °C for 5 min. Cells with arginine in ethanol vehicle were used as positive controls while untreated cells served as negative controls. The cell supernatant was collected at the end of the incubation and stored at −80 °C before nitrite and nitrate analysis. The releases of nitrite and nitrate from stimulated neutrophils were measured by stable isotope labeled GC‐MS as previously described (Tsikas 2000).

### Statistical analysis

Statistical analyses were performed using SPSS version 22.0 (SPSS Inc., Chicago, Ill., U.S.A.) and SAS version 9.4 (SAS Institute Inc., Cary, N.C., U.S.A.). For the human study data, the baseline‐adjusted between‐group differences were analyzed with random effects models with PROC MIXED (SAS) with Tukey's adjustment for multiple comparisons. In these models, subjects were treated as the random effect, and treatment, period, and treatment order were treated as the fixed effects. Correlation was analyzed using Spearman correlation analysis. Between‐group difference for *in vitro* data was analyzed using ANOVA with Bonferroni *post hoc* comparison. The results analyzed were considered to be significantly different if the *P* value was < 0.05.

## Results and Discussion

Eighteen participants (12 female, 35.3 [mean] years) completed the randomized, placebo‐controlled, cross‐over, double‐blind study (Table 1). The baseline characteristics of the participants were presented in Table 1. All participants were within the normal range of body mass index (18.5 to 25.0 kg/m^2^; Table 1). The weights, heights and body mass indices of the participants did not change during the entire course of the intervention study. All the participants were normotensive (Table 1). Their systolic and diastolic blood pressures remained unchanged throughout the 4 visits to the study center. The participants did not modify their fruits and vegetable intakes during the study.

**Table 1:** Baseline characteristics of the study participants (*n* = 18)

Age (years)^a^	35.3 (22.0, 55.0)
Gender, female	66.7%
Weight (kg)^b^	62.7 ± 13.0
Height (m)^b^	1.65 ± 0.07
Body mass index (kg/ m^2^)^b^	22.8 ± 3.7
Systolic blood pressure (mmHg)^b^	113.9 ± 11.8
Diastolic blood pressure (mmHg)^b^	68.6 ± 6.4

a Age is presented as mean (minimum, maximum).

b Weight, height, body mass index, systolic, and diastolic blood pressures were presented as mean ± SD.

**Figure 1:**
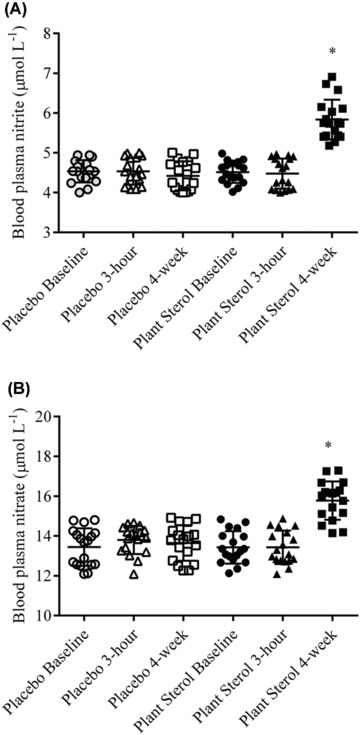
(A) Nitrite and (B) nitrate concentrations in blood plasma of 18 healthy adults before (baseline), 3 h after intervention (3 h) and after 4‐wk daily exposure (4 wk) of the placebo and plant sterols esters (2 g free plant sterols equivalent)‐enriched soy milk. ^*,^ *P* < 0.05 compared with placebo soy milk after baseline adjustment (mixed model analysis with Tukey's test).

**Figure 2:**
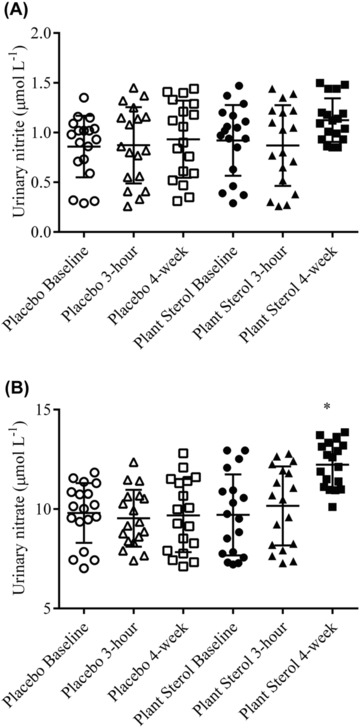
(A) Nitrite and (B) nitrate concentrations in the urine of 18 healthy adults before (baseline), 3 h after intervention (3 h) and after 4‐wk daily exposure (4 wk) of the placebo and plant sterols esters (2 g free plant sterols equivalent)‐enriched soy milk. ^*,^ *P* < 0.05 compared placebo soy milk after baseline adjustment (mixed model analysis with Tukey's test).

Circulating blood plasma nitrite and nitrate concentrations were significantly elevated after 4‐wk exposure to the plant sterols compared to the placebo concentration (Figure 1A and B). Plant sterols did not change blood plasma nitrite and nitrate concentrations 3 h after supplementation (Figure 1A and B). The urinary nitrite concentrations were unaffected by PS supplementation (Figure 2A).  The nitrate concentrations in the urine were significantly increased after 4‐wk PS treatment when compared with the placebo levels (Figure 2B). The baseline concentrations of nitrite and nitrate were comparable to previous studies (Zamani and others 2015; Zamani and others 2016). Our results showed that PS supplementation (2.0 g daily over 4 wk) augmented blood plasma nitrite and nitrate production in healthy adults, and added to the limited information about the effect of dietary PS on NO status *in vivo*. PS (2.0 g daily over 4 wk) did not restore endothelial function as measured by flow‐mediated brachial artery dilation, despite lowering the LDL cholesterol, in prepubertal children with familial hypercholesterolemia (De Jongh and others 2003). Ten‐week daily intervention with up to 1.98 g plant sterols did not change the flow‐mediated brachial artery diameter in hypercholesterolemic subjects (Hallikainen and others 2006). Concentrations of circulating E‐selectin, soluble intracellular adhesion molecule and vascular adhesion molecule‐1 were not changed after 16 wk intervention of 2.5 g PS in hypercholesterolemic subjects (De Jong and others 2007). Sitosterol and campesterol increased prostacyclin release in vascular smooth muscle cells *in vitro*, which may translate to *in vivo* vasodilation (Awad and others 2001). These previous study did not examine the effects of PS supplementation on nitrite and nitrate status *in vivo*, and the influence of their concentrations on the measured endothelial function. Plasma nitrite and nitrate have been shown to be mainly derived from the NO–arginine pathway (Rhodes and others 1995). The elevated blood plasma nitrite and nitrate concentrations in rats after nitrate‐rich beet root juice consumption was translated to augment muscle blood flow during exercise (Ferguson and others 2013). The supplemented nitrate was reduced to nitrite and NO, which preferentially increased vascular conductance and oxygen delivery to the contracting skeletal muscles in rats (Ferguson and others 2014). Inorganic nitrate (6 mmol twice daily) consumption significantly restored endothelial function in patients suffering from heart failure with Preserved Ejection Fraction and thereby improving their exercise capacity and quality of life (Zamani and others 2015, 2016). The observed blood plasma NO_2_ and NO_3_ concentrations in this study were low compared the reported result in physiological changes in human. However, there are differences in the measurement of nitrite/ nitrate where the report (Zamani and others 2015, 2016) measured NO, nitrite and nitrate, and also NO‐metal complexes and protein–NO adducts. Plasma nitrite concentrations were also found to associate with flow‐mediated dilation and thereby reflect the degree of endothelial dysfunction in a study involving 12 patients with endothelial dysfunction and 12 healthy individuals (Kleinbongard and others 2006). Recently, total serum nitrite and nitrate concentrations have been shown to be a valuable biomarker for incident cardiovascular disease (CVD), and has added value beyond traditional CVD risk factors (Hadaegh and others 2016). The elevated nitrite and nitrate levels observed in this study may be contributed by the augmented eNOS activity and the observation may be due to enhanced endothelial function. PS supplementation may improve endothelial function via the augmentation of NO production *in vivo*.

**Figure 3:**
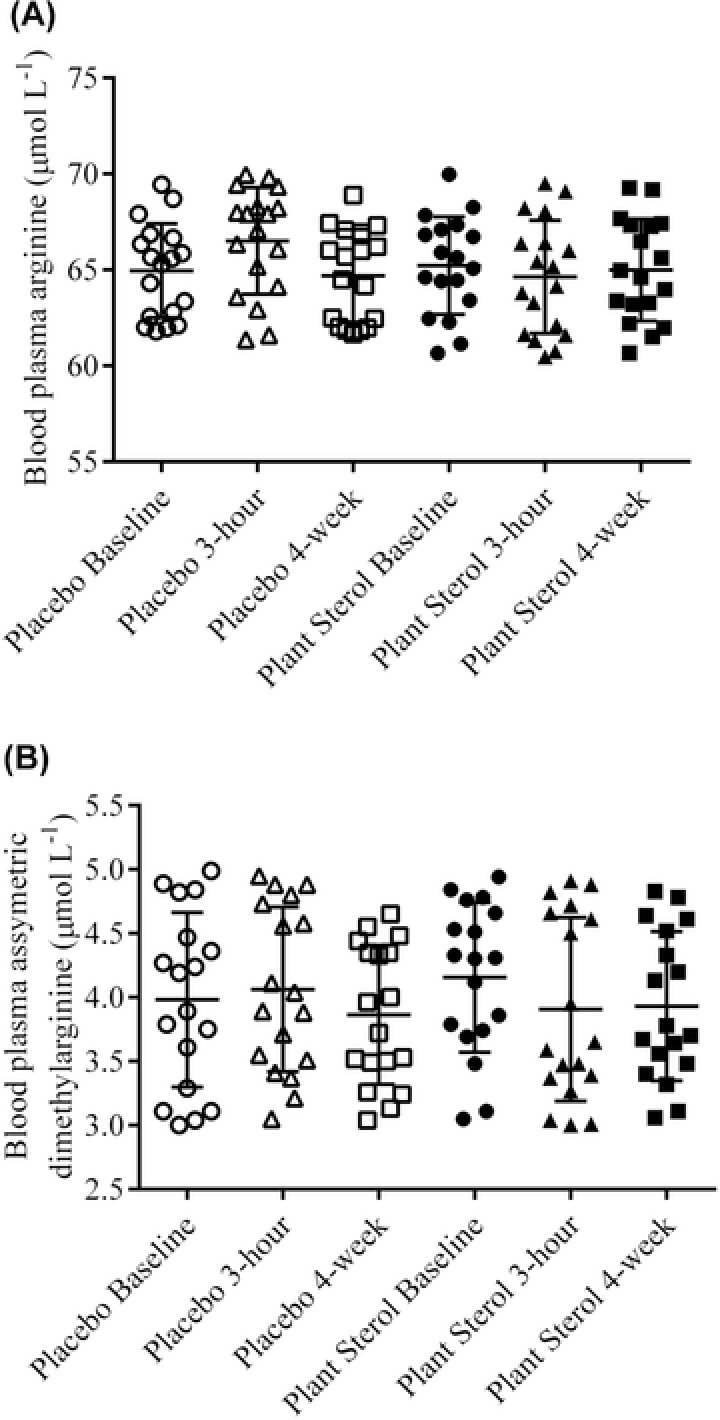
(A) Arginine and (B) asymmetric dimethylarginine concentrations in blood plasma of 18 healthy adults before (baseline), 3 h after intervention (3 h) and after 4‐wk daily exposure (4 wk) of the placebo and plant sterols esters (2 g free plant sterols equivalent)‐enriched soy milk. ^*,^ *P* < 0.05 compared placebo soy milk after baseline adjustment (mixed model analysis with Tukey's test).

The blood plasma arginine and ADMA were not affected by PS supplementation (Figure 3A and B). NO is produced from the N*‐*guanido terminal of the L‐arginine and molecular oxygenate, via the enzymatic action of eNOS in the vasculature (Maxwell and Cooke 1998). Freshly isolated human endothelial cells contained approximately 1 to 2 mmol/L L‐arginine (Li and Förstermann 2000). Because the *K*_m_ value of purified eNOS for L‐arginine is only 2.9 μmol/L (Harrison 1997), eNOS may be saturated with L‐arginine and thereby may not respond to changes in *L*‐arginine concentrations. Paradoxically, NO production was increased dose‐dependently when L‐arginine concentrations were increased in endothelial cell culture from 0.1 to 10 mmol/L (Harrison 1997). *In vivo* results agreed with the *in vitro* observation as significant associations of plasma levels of L‐arginine with enhanced vascular and systemic NO production were reported. The intracellular concentration of L‐arginine has been tightly regulated by various mechanisms, including the colonization of arginine transporter, eNOS in the membrane‐associated caveolae, its intracellular compartmentalization, arginine activity, and expression, the alteration of eNOS dimerization and competitive inhibition of eNOS by asymmetric dimethylarginine (ADMA; Nagase and others 1997; Maxwell and Cooke 1998). ADMA, a naturally‐occurring modified amino acid in human blood, inhibits all 3 NOS isoforms (Kielstein and others 2007), and thereby reduces the production of NO *in vivo* (Leone and others 1992). The levels of ADMA had been associated with flow‐mediated dilatation (Ardigo and others 2007; Päivä and others 2010). A recent meta‐analysis of available prospective studies suggested associations between circulating ADMA concentrations and cardiovascular disease outcomes under a broad range of circumstances (Willeit and others 2015). The increase in nitrite and nitrate circulating concentrations were not accompanied by changes in the L‐arginine and ADMA levels, suggesting that the PS modulates NO production and hypothetically endothelial function, independently of L‐arginine and ADMA.

Our results on the bioavailability of PS had been previously presented and discussed (Ho and others 2016). Serum concentrations of total PS increased after 3 h and 4‐wk daily exposure to 2.0 g PS daily when compared to the placebo concentrations (Ho and others 2016). The specific PS (β‐sitosterol, campesterol, and stigmasterol) concentrations in blood plasma and urine were significantly elevated after 4‐wk PS treatments when compared to placebo concentrations (Ho and others 2016). After 4‐wk PS supplementation, the nitrite and nitrate concentrations in blood plasma correlated significantly to the plasma total PS (nitrite *R* = 0.64, *P* < 0.05; nitrate *R* = 0.42, *P* < 0.05) and specific PS (nitrite: β*‐*sitosterol, *r* = 0.80, *P* < 0.05; nitrate: *β*‐sitosterol, *r* = 0.77, *P* < 0.05; nitrite: campesterol, *r* = 0.63, *P* < 0.05; nitrate: campesterol, *r* = 0.62, *P* < 0.05; nitrite: stigmasterol, *r* = 0.50, *P* < 0.05; nitrate: stigmasterol, *r* = 0.71, *P* < 0.05) concentrations. These correlations were absent with the 3‐h PS treatment and all stages of the placebo treatment. Urinary nitrite and nitrate concentrations did not correlate to the plasma total and specific PS concentrations. Arginine and ADMA concentrations in the blood plasma were not associated with the total and specific PS concentrations. The significant correlations between the nitrite, nitrate, and specific PS concentrations in the blood circulation observed in our study further support the suggested augmentation of NO production by the PS. The absence of the 3‐h effects in our study may be explained by choice of the NO markers. As nitrite and nitrate are formed by the downstream oxidation of NO, their measurements may not truly reflect the NO status at the specific time point.

**Figure 4:**
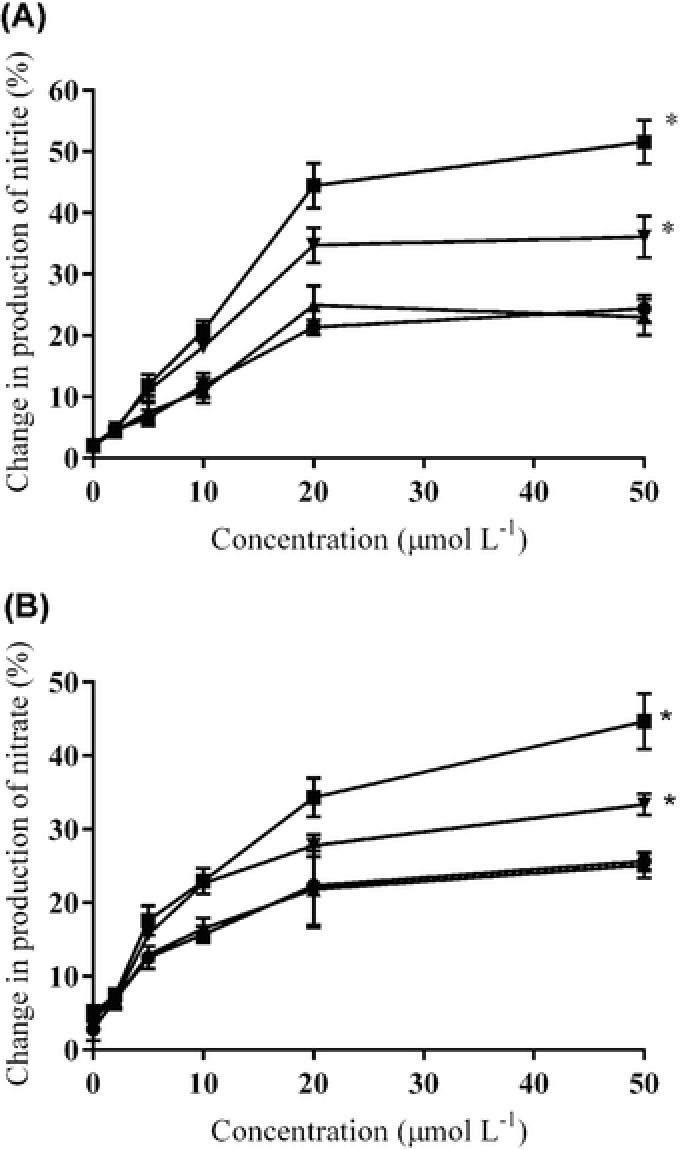
Changes (%) in (A) nitrite and (B) nitrate formation by phorbol 12‐myristat 13‐acetate‐stimulated human blood neutrophils treated with β‐sitosterol (●), campesterol (■), stigmasterol (▲) and plant sterols mixture (50% β‐sitosterol, 30% campesterol, 20% stigmasterol w/w, ▼; 0 to 50 μmol/L) compared to the untreated phorbol 12‐myristat 13‐acetate‐stimulated human blood neutrophils. ^*,^ *P* < 0.05 compared with others using ANOVA of AUC with Bonferroni *post hoc* adjustment.

The effects of specific PS and PS mixture on the total nitrite and nitrate production from the activated human blood neutrophils were expressed as the percentage increase in the total nitrite and nitrate production relative to the positive controls. All 3 specific PS increased nitrite and nitrate production from the neutrophils(*P* < 0.05 compared with positive control using ANOVA of AUC, *n* = 5; Figure 4A and B). Campesterol augmented nitrite and nitrate formation to a significant extent than *β*‐sitosterol, stigmasterol, and plant sterol mixture (*P* < 0.05 using ANOVA of AUC, *n* = 5; Figure 4A and B). The PS mixture significantly increased nitrite and nitrate production compared to β‐sitosterol and stigmasterol (*P* < 0.05 using ANOVA of AUC, *n* = 5; Figure 4A and B). A significant difference was absent between β‐sitosterol and stigmasterol (*P* < 0.05 using ANOVA of AUC, *n* = 5; Figure 5A and B). Specific PS, such as β‐sitosterol, campesterol, and stigmasterol exerted differential effects on the nitrite and nitrate production *in vitro*. The more vasoactive campesterol was naturally present at significantly lower proportion than the less active β‐sitosterol, as our daily diet is typically made up of 65% β‐sitosterol, 30% campesterol, and 5% stigmasterol (Ling and Jones 1995). Our *in vitro* results support the incorporation or enrichment of PS mixture or specific PS, like campesterol, into suitable food products to further enhance their vasoprotective benefits. Our *in vivo* findings were supported by our *in vitro* results where the productions of nitrite and nitrate were increased in the PS‐treated cells. The *in vitro* results were made more physiological relevant when the specific PS were loaded at physiological achievable concentrations into the cells.

Our study is limited to the measurement of vascular NO pathway. It did not measure flow‐mediated dilation and other markers of endothelial function in responses to the PS supplementation. More studies are required to examine the effects of dietary PS on specific markers of endothelial function.

## Conclusion

PS supplementation in the presence of a food matrix may upregulate the vascular NO production.

## Authors’ Contributions

WML designed research; XLH and WML conducted experiments; XLH and WML analyzed data; and WML wrote the paper. WML had primary responsibility for final content. All authors read and approved the final manuscript.

## References

[jfds13752-bib-0001] AbuMweis SS , Barake R , Jones PJH . 2008 Plant sterols/stanols as cholesterol lowering agents: a meta‐analysis of randomized controlled trials. Food Nutr Res 52(1):1811–24. 10.3402/fnr.v52i0.1811 PMC2596710 19109655

[jfds13752-bib-0002] Ahmida HSM , Bertucci P , Franzò L , Massoud R , Cortese C , Lala A , Federici G . 2006 Simultaneous determination of plasmatic phytosterols and cholesterol precursors using gas chromatography–mass spectrometry (GC–MS) with selective ion monitoring (SIM). J Chromatogr B 842(1):43–7. 10.1016/j.jchromb.2006.05.024 16807145

[jfds13752-bib-0003] Ardigo D , Stüehlinger M , Franzini L , Valtuena S , Piatti P , Pachinger O , Reaven G , Zavaroni I . 2007 ADMA is independently related to flow‐mediated vasodilation in subjects at low cardiovascular risk. Eur J Clin Invest 37(4):263–9. 17373961 10.1111/j.1365-2362.2007.01781.x

[jfds13752-bib-0004] Awad AB , Smith AJ , Fink CS . 2001 Plant sterols regulate rat vascular smooth muscle cell growth and prostacyclin release in culture. Prostagl Leukotr Essent Fatty Acids 64(6):323–30. 10.1054/plef.2001.0273 11427042

[jfds13752-bib-0005] Chan Y‐M , Demonty I , Pelled D , Jones PJ . 2007 Olive oil containing olive oil fatty acid esters of plant sterols and dietary diacylglycerol reduces low‐density lipoprotein cholesterol and decreases the tendency for peroxidation in hypercholesterolaemic subjects. Br J Nutr 98(3):563–70. 17559697 10.1017/S0007114507730775

[jfds13752-bib-0006] Clarkson P , Adams MR , Powe AJ , Donald AE , McCredie R , Robinson J , McCarthy SN , Keech A , Celermajer DS , Deanfield JE . 1996 Oral L‐arginine improves endothelium‐dependent dilation in hypercholesterolemic young adults. J Clin Invest 97(8):1989–94. 8621785 10.1172/JCI118632 PMC507270

[jfds13752-bib-0007] De Jong A , Plat J , Bast A , Godschalk RWL , Basu S , Mensink RP . 2007 Effects of plant sterol and stanol ester consumption on lipid metabolism, antioxidant status and markers of oxidative stress, endothelial function and low‐grade inflammation in patients on current statin treatment. Eur J Clin Nutr 62(2):263–73. 17487211 10.1038/sj.ejcn.1602733

[jfds13752-bib-0008] De Jongh S , Vissers M , Rol P , Bakker H , Kastelein J , Stroes E . 2003 Plant sterols lower LDL cholesterol without improving endothelial function in prepubertal children with familial hypercholesterolaemia. J Inherit Metabol Dis 26(4):343–52. 10.1023/a:1025155002348 12971422

[jfds13752-bib-0009] Demonty I , Ras RT , van der Knaap HCM , Duchateau GSMJE , Meijer L , Zock PL , Geleijnse JM , Trautwein EA . 2008 Continuous dose‐response relationship of the LDL‐cholesterol–lowering effect of phytosterol intake. J Nutr 139(2):1–10. 19091798 10.3945/jn.108.095125

[jfds13752-bib-0010] Ferguson SK , Hirai DM , Copp SW , Holdsworth CT , Allen JD , Jones AM , Musch TI , Poole DC . 2013 Impact of dietary nitrate supplementation via beetroot juice on exercising muscle vascular control in rats. J Physiol 591(2):547–57. 23070702 10.1113/jphysiol.2012.243121 PMC3577528

[jfds13752-bib-0011] Ferguson SK , Hirai DM , Copp SW , Holdsworth CT , Allen JD , Jones AM , Musch TI , Poole DC . 2014 Dose dependent effects of nitrate supplementation on cardiovascular control and microvascular oxygenation dynamics in healthy rats. Nitric Oxide 39:51–8. 24769046 10.1016/j.niox.2014.04.007 PMC4109815

[jfds13752-bib-0012] Gladwin MT , Raat NJ , Shiva S , Dezfulian C , Hogg N , Kim‐Shapiro DB , Patel RP . 2006 Nitrite as a vascular endocrine nitric oxide reservoir that contributes to hypoxic signaling, cytoprotection, and vasodilation. Am J Physiol ‐ Heart Circ Physiol 291(5):H2026–H35. 16798825 10.1152/ajpheart.00407.2006

[jfds13752-bib-0013] Hadaegh F , Asgari S , Bozorgmanesh M , Jeddi S , Azizi F , Ghasemi A . 2016 Added value of total serum nitrate/nitrite for prediction of cardiovascular disease in middle east caucasian residents in Tehran. Nitric Oxide 54:60–6. 26923817 10.1016/j.niox.2016.02.004

[jfds13752-bib-0014] Hallikainen M , Lyyra‐Laitinen T , Laitinen T , Ågren JJ , Pihlajamäki J , Rauramaa R , Miettinen TA , Gylling H . 2006 Endothelial function in hypercholesterolemic subjects: effects of plant stanol and sterol esters. Atherosclerosis 188(2):425–32. 16386259 10.1016/j.atherosclerosis.2005.11.012

[jfds13752-bib-0015] Harrison DG . 1997 Cellular and molecular mechanisms of endothelial cell dysfunction. J Clin Invest 100(9):2153. 9410891 10.1172/JCI119751 PMC508409

[jfds13752-bib-0016] Ho X , Tsen SY , Ng MY , Lee WN , Low A , Loke WM . 2016 Aged garlic , not raw garlic precursor, supplement protects against lipid peroxidation in hypercholesterolemic individuals. J Med Food 19:931–7. 10.1089/jmf.2016.3693 27627579

[jfds13752-bib-0017] Hu FB , Willett WC . 2002 Optimal diets for prevention of coronary heart disease. J Am Med Assoc 288(20):2569–78. 10.1001/jama.288.20.2569 12444864

[jfds13752-bib-0018] Kielstein A , Tsikas D , Galloway GP , Mendelson JE . 2007 Asymmetric dimethylarginine (ADMA)—a modulator of nociception in opiate tolerance and addiction? Nitric Oxide 17(2):55–9. 17625935 10.1016/j.niox.2007.05.005 PMC2025594

[jfds13752-bib-0019] Kleinbongard P , Dejam A , Lauer T , Jax T , Kerber S , Gharini P , Balzer J , Zotz RB , Scharf RE , Willers R , Schechter AN , Feelisch M , Kelm M . 2006 Plasma nitrite concentrations reflect the degree of endothelial dysfunction in humans. Free Rad Biol Med 40(2):295–302. 16413411 10.1016/j.freeradbiomed.2005.08.025

[jfds13752-bib-0020] Kleinbongard P , Dejam A , Lauer T , Rassaf T , Schindler A , Picker O , Scheeren T , Gödecke A , Schrader J , Schulz R , Heusch G , Schaub GA , Bryan NS , Feelisch M , Kelm M . 2003 Plasma nitrite reflects constitutive nitric oxide synthase activity in mammals. Free Rad Biol Med 35(7):790–6. 14583343 10.1016/s0891-5849(03)00406-4

[jfds13752-bib-0021] Lau VW , Journoud M , Jones PJ . 2005 Plant sterols are efficacious in lowering plasma LDL and non‐HDL cholesterol in hypercholesterolemic type 2 diabetic and nondiabetic persons. Am J Clin Nutr 81(6):1351–8. 15941886 10.1093/ajcn/81.6.1351

[jfds13752-bib-0022] Leone A , Moncada S , Vallance P , Calver A , Collier J . 1992 Accumulation of an endogenous inhibitor of nitric oxide synthesis in chronic renal failure. Lancet 339(8793):572–5. 1347093 10.1016/0140-6736(92)90865-z

[jfds13752-bib-0023] Li H , Förstermann U . 2000 Nitric oxide in the pathogenesis of vascular disease. J Pathol 190(3):244–54. 10685059 10.1002/(SICI)1096-9896(200002)190:3<244::AID-PATH575>3.0.CO;2-8

[jfds13752-bib-0024] Ling WH , Jones PJH . 1995 Dietary phytosterols: a review of metabolism, benefits, and side effects. Life Sci 57(3):195–206. 7596226 10.1016/0024-3205(95)00263-6

[jfds13752-bib-0025] Loke WM , Lam KM‐J , Chong WL , Chew SE , Quek AM , Lim EC , Seet RC . 2012 Products of 5‐lipoxygenase and myeloperoxidase activities are increased in young male cigarette smokers. Free Rad Res 46(10):1230–7. 10.3109/10715762.2012.701291 22690830

[jfds13752-bib-0026] Maxwell AJ , Cooke JP . 1998 Cardiovascular effects of *L*‐arginine. Curr Opin Nephol Hypertens 7(1):63–70. 10.1097/00041552-199801000-00011 9442365

[jfds13752-bib-0027] Nagase S , Takemura K , Ueda A , Hirayama A , Aoyagi K , Kondoh M , Koyama A . 1997 A novel nonenzymatic pathway for the generation of nitric oxide by the reaction of hydrogen peroxide and D‐or L‐arginine. Biochem Biophys Res Comm 233(1):150–3. 9144413 10.1006/bbrc.1997.6428

[jfds13752-bib-0028] Ostlund RE , McGill JB , Zeng C‐M , Covey DF , Stearns J , Stenson WF , Spilburg CA . 2002 Gastrointestinal absorption and plasma kinetics of soy Δ5‐phytosterols and phytostanols in humans. Am J Physiol Endocrinol Metab 282(4):E911–E6. 11882512 10.1152/ajpendo.00328.2001

[jfds13752-bib-0029] Päivä H , Kähönen M , Lehtimäki T , Alfthan G , Viikari J , Laaksonen R , Hutri‐Kähönen N , Laitinen T , Taittonen L , Raitakari OT . 2010 Levels of asymmetrical dimethylarginine are predictive of brachial artery flow‐mediated dilation 6 years later. The Cardiovascular Risk in Young Finns Study. Atherosclerosis 212(2):512–5. 20655043 10.1016/j.atherosclerosis.2010.06.041

[jfds13752-bib-0030] Rassaf T , Preik M , Kleinbongard P , Lauer T , Heiß C , Strauer B‐E , Feelisch M , Kelm M . 2002 Evidence for *in vivo* transport of bioactive nitric oxide in human plasma. J Clin Invest 109(9):1241–8. 11994413 10.1172/JCI14995 PMC150967

[jfds13752-bib-0031] Rhodes PM , Leone AM , Francis PL , Struthers AD , Moncada S . 1995 The L‐Arginine: Nitric oxide pathway is the major source of plasma nitrite in fasted humans. Biochem Biophys Res Comm 209(2):590–6. 7794389 10.1006/bbrc.1995.1541

[jfds13752-bib-0032] Thorne S , Mullen MJ , Clarkson P , Donald AE , Deanfield JE . 1998 Early endothelial dysfunction in adults at risk from atherosclerosis: different responses to *L*‐arginine. J Am Coll Cardiol 32(1):110–6. 9669257 10.1016/s0735-1097(98)00211-3

[jfds13752-bib-0033] Tsikas D . 2000 Simultaneous derivatization and quantification of the nitric oxide metabolites nitrite and nitrate in biological fluids by gas chromatography/mass spectrometry. Anal Chem 72(17):4064–72. 10994966 10.1021/ac9913255

[jfds13752-bib-0034] Verma S , Anderson TJ . 2002 Fundamentals of endothelial function for the clinical cardiologist. Circulation 105(5):546–9. 11827916 10.1161/hc0502.104540

[jfds13752-bib-0035] Vita JA , Keaney JF . 2002 Endothelial function a barometer for cardiovascular risk? Circulation 106(6):640–2. 12163419 10.1161/01.cir.0000028581.07992.56

[jfds13752-bib-0036] Willeit P , Freitag DF , Laukkanen JA , Chowdhury S , Gobin R , Mayr M , Di Angelantonio E , Chowdhury R . 2015 Asymmetric dimethylarginine and cardiovascular risk: systematic review and meta‐analysis of 22 prospective studies. J Am Heart Assoc 4(6):e001833. 26021436 10.1161/JAHA.115.001833 PMC4599532

[jfds13752-bib-0037] Zamani P , Rawat D , Shiva‐Kumar P , Geraci S , Bhuva R , Konda P , Doulias P‐T , Ischiropoulos H , Townsend RR , Margulies KB , Cappola TP , Poole DC , Chirinos JA . 2015 Effect of inorganic nitrate on exercise capacity in heart failure with preserved ejection fraction. Circulation 131(4):371–80. 25533966 10.1161/CIRCULATIONAHA.114.012957 PMC5823250

[jfds13752-bib-0038] Zamani P , Tan VX , Soto‐Calderon H , Beraun M , Brandimarto J , Trieu L , Varakantam S , Doulias P‐T , Townsend RR , Chittams J , Margulies KB , Cappola TP , Poole DC , Ischiropoulos H , Chirinos JA . 2016 Pharmacokinetics and pharmacodynamics of inorganic nitrate in heart failure with preserved ejection fraction. Circulation 134:A15751. 10.1161/CIRCRESAHA.116.309832 PMC5376233 27927683

